# Dimerization misalignment in human glutamate-oxaloacetate transaminase variants is the primary factor for PLP release

**DOI:** 10.1371/journal.pone.0203889

**Published:** 2018-09-12

**Authors:** Jesi Lee, Trevor Gokey, Dylan Ting, Zheng-Hui He, Anton B. Guliaev

**Affiliations:** 1 Department of Chemistry and Biochemistry, San Francisco State University, San Francisco, CA, United States of America; 2 Department of Biology, San Francisco State University, San Francisco, CA, United States of America; Weizmann Institute of Science, ISRAEL

## Abstract

The active form of vitamin B6, pyridoxal 5’-phosphate (PLP), plays an essential role in the catalytic mechanism of various proteins, including human glutamate-oxaloacetate transaminase (hGOT1), an important enzyme in amino acid metabolism. A recent molecular and genetic study showed that the E266K, R267H, and P300L substitutions in aspartate aminotransferase, the *Arabidopsis* analog of hGOT1, genetically suppress a developmentally arrested *Arabidopsis RUS* mutant. Furthermore, CD analyses suggested that the variants exist as apo proteins and implicated a possible role of PLP in the regulation of PLP homeostasis and metabolic pathways. In this work, we assessed the stability of PLP bound to hGOT1 for the three variant and wildtype (WT) proteins using a combined 6 *μs* of molecular dynamics (MD) simulation. For the variants and WT in the holo form, the MD simulations reproduced the “closed-open” transition needed for substrate binding. This conformational transition was associated with the rearrangement of the P15-R32 small domain loop providing substrate access to the R387/R293 binding motif. We also showed that formation of the dimer interface is essential for PLP affinity to the active site. The position of PLP in the WT binding site was stabilized by a unique hydrogen bond network of the phosphate binding cup, which placed the cofactor for formation of the covalent Schiff base linkage with K259 for catalysis. The amino acid substitutions at positions 266, 267, and 300 reduced the structural correlation between PLP and the protein active site and/or integrity of the dimer interface. Principal component analysis and energy decomposition clearly suggested dimer misalignment and dissociation for the three variants tested in our work. The low affinity of PLP in the hGOT1 variants observed in our computational work provided structural rationale for the possible role of vitamin B6 in regulating metabolic pathways.

## Introduction

Vitamin B6 is essential to all living organisms. It serves as a cofactor in metabolic pathways and is a potent antioxidant against stress [1–3]. As a versatile cofactor affecting almost all cellular processes, vitamin B6 is a key component that regulates major metabolic decisions, and its deficiency is fatal. How an organism monitors vitamin B6 homeostasis and makes developmental decisions is currently not known. Pyridoxal 5’-phosphate (PLP), an active form of vitamin B6, is a ubiquitous cofactor for numerous enzymes in various catalytic mechanisms such as decarboxylation, racemization, transamination, α/β elimination, and retro-aldol cleavage [4, 5]. This versatile cofactor forms a covalent bond with a catalytic lysine in all PLP-dependent enzymes forming an internal aldimine, commonly known as a Schiff base. As an electrophilic catalyst, PLP stabilizes carbanionic intermediates. Deprotonation of the substrate NH_3_^+^ leads to an attack on the Schiff base carbon and the amino group replaces the ε-amino group of the lysine residue, resulting in an external aldimine [6, 7]. The mechanism of the external aldimine formation from a Schiff base is a conserved mechanistic feature in all PLP-catalyzed reactions. Once this common central intermediate is formed, the reaction proceeds depending on the enzyme’s function [8].

Aspartate aminotransferase (AAT), the most studied PLP-dependent enzyme, has provided much mechanistic information, such as Schiff base and external aldimine formation and the role of its cofactor PLP [9]. AAT has a highly conserved sequence throughout species, including non-human forms of AAT of various species ranging from E.coli to plants, chicken, and pig [10, 11]. There is high sequence similarity between hGOT1 and *Arabidopsis* AAT (68.8%). This class of enzymes is particularly well-conserved within the active site (92%). It is also been previously reported that different species have essentially the same secondary structure for this enzyme [12, 13]. The human form of AAT, cytoplasmic glutamate oxaloacetate aminotransferase (hGOT1), has been crystallized only recently (PDB ID 3II0) [14]. hGOT1 catalyzes the reversible conversion of aspartate and α-ketoglutarate to oxaloacetate and glutamate in amino acid metabolism and is found in tissues of liver, heart, skeletal muscle, brain, and red blood cells. The enzyme is commonly used as a clinical marker since the serum level of hGOT1 indicates the severity of damaged liver and heart tissue [15]. hGOT1 expression correlates with tumor growth due to use of the glutamine by various cancer cells. Moreover, it has been proposed that inhibition of aspartate aminotransferase, which acts together with malate dehydrogenase to transfer electrons in mitochondria, could disrupt the growth and metabolism of breast adenocarcinoma cells [16, 17].

**Fig 1:**
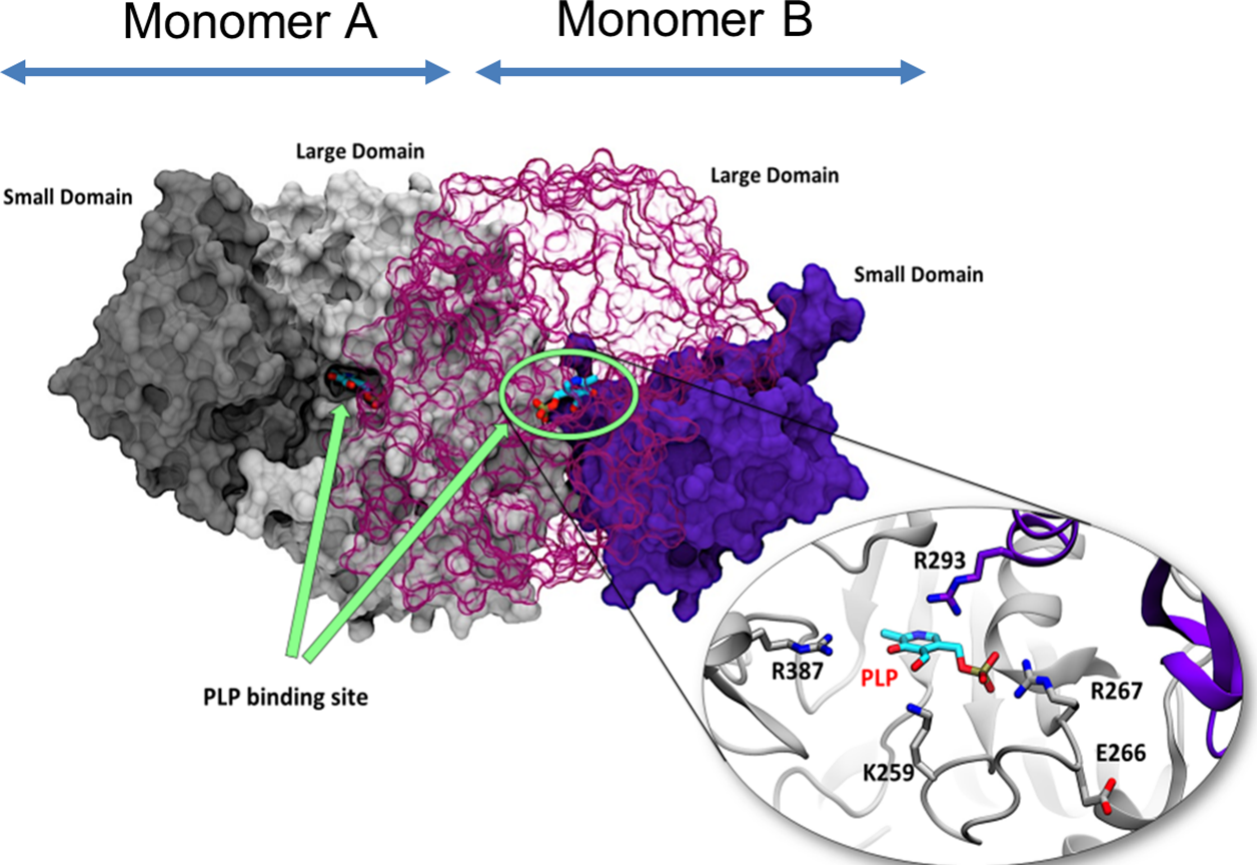
Structural representation of wild-type hGOT1 based on crystallographic coordinates PDB ID 3II0.

Structurally, hGOT1 is characterized as a homodimeric (α_2_) enzyme where each monomer consists of a large and small domain (Fig 1). The small domain of the enzyme is shaped by four α-helices, three β-strands, and the N- and C- termini. The large domain consists of a 7-stranded β-sheet and several short α helices, which we refer to as the large domain core. The large domains from each monomer form the dimer interface, and two PLP binding sites are located at this interface. PLP is stabilized by surrounding residues, where the D223 residue acts as an anchor by forming a hydrogen bond with the positively charged nitrogen in the pyridine of PLP. The indole ring of W141 participates in both π-π aromatic stacking and hydrophobic interactions with the pyridine of PLP. The phosphate group of PLP is stabilized by G109, T110, S256, S258, and R267 of the same monomer as well as T71 from the opposite monomer. The two primary substrate binding residues are R387 and R293 and are located at the entrance of the binding site (Fig 1, inset view).

hGOT1 is a homodimer where each monomer, labeled A and B, comprises a small and large domain (PDB ID 3II0). The small and large domains of monomer A are colored in dark and light grey respectively. The small and large domains of monomer B are colored dark and light purple respectively. Two identical PLP binding sites are located at the dimeric interface between the small and large domains of each monomer (indicated by green arrows). The close-up view of the active site shows catalytically important residues, including K259 essential for the formation of the PLP-K259 aldimine and the R387/R293 substrate binding motif.

Recent biological studies of a plant model, *Arabidopsis thaliana*, revealed the importance of PLP in plant morphogenesis [18–20]. Like the cells in any other sun-exposed organism, plant cells must cope with a certain amount of ultraviolet-B (UV-B) radiation (280–320 nm) in order to survive. PLP is known to be highly susceptible to UV-B and is irreversibly converted to a photoproduct when exposed to UV light for 5 min. When wild-type *Arabidopsis* absorbs UV-B, the *RUS1/RUS2* (Root UV-B Sensitive 1 and 2) complex plays a role in safeguarding PLP homeostasis. However, when *RUS1* or *RUS2* is mutated, disturbances in PLP homeostasis results in total developmental block [12, 20–22]. Further analysis showed that specific amino acid substitutions in AAT genetically suppressed the *RUS1/RUS2* mutant and exhibited the same effect as adding vitamin B6 exogenously, suggesting AAT may play a critical role in PLP homeostasis regulation [20]. Currently, there is no crystal structure of *Arabidopsis* AAT. The three specific mutations in AAT that rescued *RUS* mutants in *Arabidopsis* and displayed the most growth were E258K, R259H and P292L. Although these mutations are located at different positions in the enzyme, near-UV CD spectrum experiments indicated that wild-type AAT had PLP bound in its pockets, whereas the variants showed little sign of cofactor binding. These data demonstrated that cofactors in the mutated enzymes may be released as a free-floating form resulting in a suppressed *RUS* mutant and healthy plant growth, suggesting that a developmental signaling pathway in the plant model may be influenced by vitamin B6 homeostasis. While a constant PLP supply is required for normal cellular metabolic function, excess free PLP can be toxic as it can non-specifically form covalent bonds with both thiol and amino groups [23, 24]. How cells monitor and maintain PLP homeostasis is currently not understood. Previous studies suggested that AAT homologs could have similar behavior as *Arabidopsis* AAT in its possible role in PLP homeostasis [20].

Though we cannot directly compare the role of the free PLP in plant homeostasis to humans, there is mounting evidence that vitamin B6 is associated with carcinogenic events caused by UV exposure in mammals, including humans. Photosensitivity has been shown in both *in-vivo* and *in-vitro* models after treatment with excess vitamin B6 [25–27]. To investigate whether the conserved residues in hGOT1 play a similar role to that of *Arabidopsis* AAT, we employed molecular dynamics (MD) to test the affinity of PLP binding in hGOT1 and the three variants: E266K, R267H, and P300L. These are equivalent to E258, R259 and P292 previously tested in *Arabidopsis* and mutations at these locations result in PLP release [20]. Given that these mutations can release PLP in the plant model, we investigated whether a similar event could occur in humans. We propose that conformational dynamics and/or energetics of variants are significantly different from a WT control. The analysis of the conformational data should reveal key aspects of variant instability and how hGOT1 generally behaves at the molecular level.

## Materials and methods

The crystal structure of glutamate oxaloacetate transaminase (hGOT1; PDB ID 3II0), the human homolog of AAT, was used as a starting structure in the simulations. This structure is the wild-type (WT) holoprotein that does not have a covalent linkage between K259 and the PLP cofactor. Five systems, including WT, E226K, R267H, P300L, and WT_monomer_ with PLP bound were prepared for molecular dynamics (MD) simulations with the AMBER molecular modeling package [28] and post-processed with implicit solvent to determine energies. The monomer energy EM represents the combined energy of the two monomers in the dimer (EM1 + EM2). The dimerization energy (EE) represents the interaction energies between the two monomers in the dimer, and the PLP binding free energy (EL) calculations were based on procedures used by us and others [29–33]. The EL energies were calculated as the sum ofboth PLP binding sites. In addition, we simulated WT with the natural substrate aspartate bound to act as a control to assess the dynamics of the loops. As a result, a total of 6 systems were used in this work. Each system was solvated in TIP3 water, using a 10 Å periodic water box and counter ions added to neutralize the overall charge. The dimensions of each box were approximately 109 x 100 x 93 Å^3^ for the complex systems, with each being about 12,800 atoms. A total of 6 μs of combined MD simulations were collected (1 μs for each complex). The last 800 ns were used to calculate the binding and interaction energies for each complex. The calculations utilized the Ewald summation method to handle long-range electrostatics with a 10 Å cutoff [34]. For temperature control, the Berendsen thermostat was used with a 1 ps velocity rescaling interval. Initially, each system was minimized with a 20 kcal/mol restraint on the protein to allow the water to relax, followed by another minimization with the restraint lifted. The systems were then heated to 300 K over 30 ps. After heating, the water was allowed to equilibrate to the proper density using a restraint of 20 kcal/mol for 200 ps. Then, after 500 ps, the restraint was lifted, and the density allowed to level out. Finally, each system was subjected to a 1000 ns production phase using a 2 fs time step under the NPT ensemble at 300 K.

Parameters for the active form of vitamin B6, pyridoxal 5'phosphate (PLP), and aspartate were generated using the B3LYP hybrid DFT model with the aug-cc-pVDZ basis set using Merz-Singh-Kollman charges in the Gaussian 09 package [35]. Though the Schiff base was not explicitly modeled, these charges along with the force-field parameters maintained close contact with K259 during the WT simulations. Electrostatic grids were then generated in Gaussian 09 from the refined structures followed by the RESP method in antechamber for charge fitting [28]. The binding energies from the simulations were sampled every one ns and post-processed with the Generalized Born implicit solvation method [36]. The OBC2 implicit water model was used with default parameters as the implicit solvent (igb = 5 in AMBER), using the LCPO surface area definition (gbsa = 1 in AMBER) [37, 38]. The remaining energy terms were calculated without periodic boundary conditions, with no cutoff for non-bonded interactions.

Principal component analysis (PCA), or essential dynamics (ED) in the MD simulation vernacular, is a useful tool for analyzing the overall correlated motions of hGOT1 [39]. The model, corresponding to the average coordinates and principal components, was calculated using simulation frames from the last 800 ns of four systems (WT, E266K, R267H, and P300L) every one ns, resulting in a total of 3,200 frames. Only the Cα atoms of residues 40 to 403 of both monomers and the C6 carbon of PLP were included, leaving out residues at both N- and C-termini to prevent our model from incorporating random fluctuations. This produced a 3Nx3N covariance matrix, where N = 730 is the number of Cα atoms. This matrix was processed using eigen-decomposition to generate the 3N PC vectors, sorted by the magnitude of their motion (the eigenvalues). The displacement along the first PC (PC1) was calculated separately for each of the four simulations to determine their relative displacement versus WT. The average displacement of WT was set to zero, and the variant displacements were analyzed relative to WT. Displacement of each snapshot can be calculated using the inner product between PC1 and the coordinates of the atoms, with PC1 being a 3Nx1 basis vector and the frame being a 1x3N coordinate vector, producing a scalar displacement value.

## Results and discussion

### Loop dynamics of human GOT1

**Fig 2:**
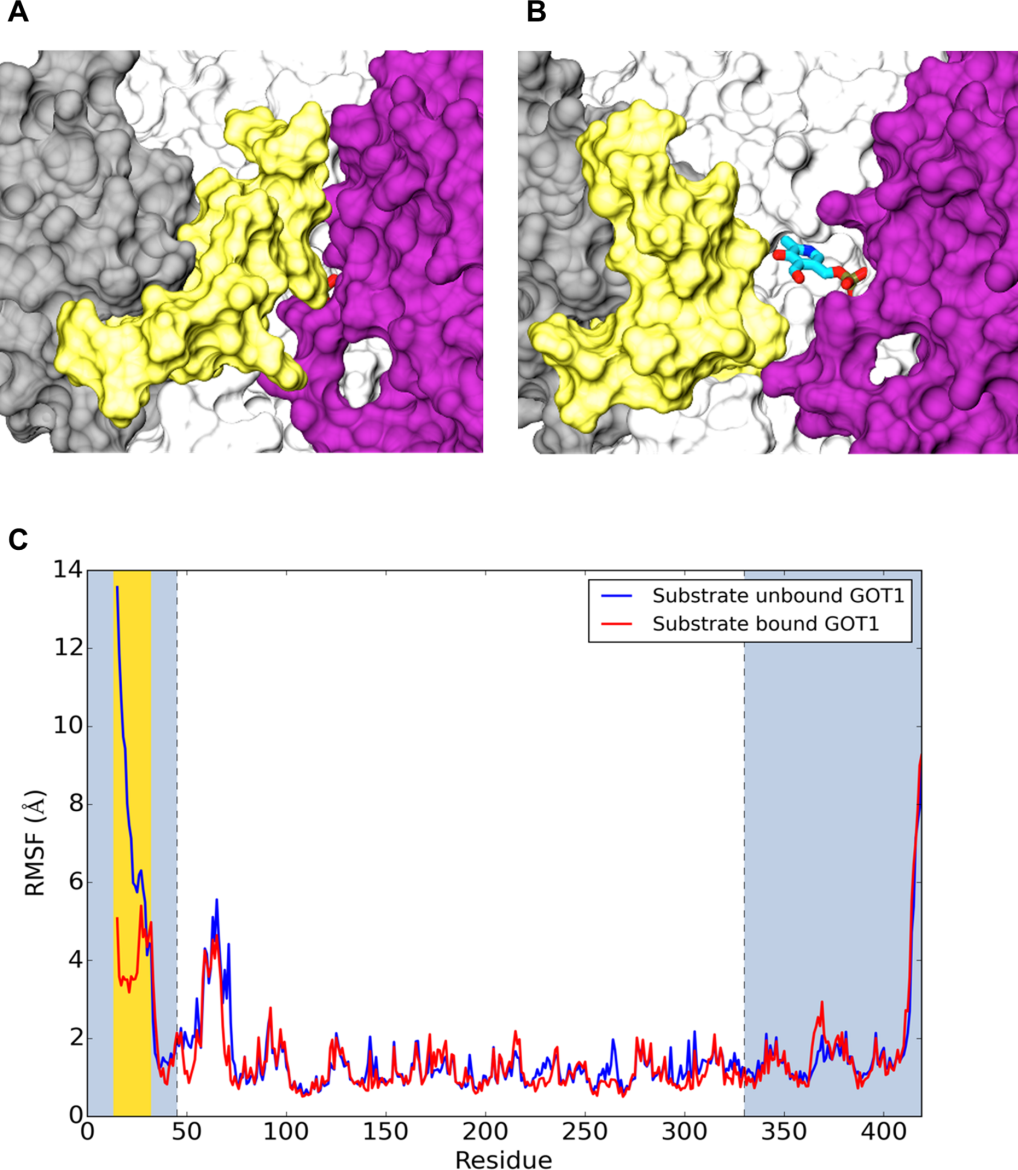
(A) Closed and (B) open states of hGOT1 produced by 1 μs MD simulation. The cofactor is represented by sticks and colored by atom, and the P15-R32 “door” is shown in yellow. The color scheme of the protein corresponds to Fig 1. (C) RMSF calculations for the substrate bound and unbound simulations of hGOT1. Both monomers A and B were averaged in RMSF analysis. The blue plot represents the simulations of WT without the substrate (unbound form) whereas the red plot describes simulation of the enzyme with substrate bound. The large domain is in white, the small domain is in gray, and the loop residues are highlighted in yellow.

The crystal structure of wild-type hGOT1 (PDB ID 3II0), used as a starting point in our simulations, contains an inhibitor, tartrate. Tartrate, similar to natural substrates, forms stable hydrogen bond interactions with R387 of the same monomer and R293 from the opposite monomer, which helps to lock the two domains of both monomer together. These interactions produced a closed conformation of the hGOT1-substrate complex without any visible access to the binding pocket. The MD simulations confirmed the stability of the closed conformation in the presence of either tartrate or the natural substrate for hGOT1, aspartate. However, when these ligands were removed from the complexes, the MD simulations revealed a closed to open conformational transition within the first 100 ns of simulation. The lack of hydrogen bonding between the ligand and the protein allowed a conformational rearrangement of the P15-R32 loop in the small domain, acting as a “door” to the enzyme active site. This loop is a part of the N-terminal *α*-helices of the small domain, which makes contact with the large domains of both monomers in the presence of substrate. The open and closed conformations of the hGOT1 active site produced by MD simulations are shown in Fig 2A and 2B.

In order to quantify the loop dynamics, we evaluated the Root Mean Square Fluctuation (RMSF) of each Cα atom as a function of residue number for hGOT1 with and without the substrate (Fig 2C). A substantial increase in the RMSF could be observed for the region between residues 15 to 32 for substrate-unbound GOT1 (blue plot, Fig 2C) compared to the substrate-bound form (red plot, Fig 2C). The absence of the substrate RMSF values for the loop residues increased by at least 10 Å, which corresponds to a 10 Å displacement (opening) of the P15-R32 loop. The opening of the P15-R32 “door” provided unobstructed access to the enzyme active site. Both simulations also revealed noticeable RMSF fluctuations for residues ranging from 58 to 72 and 413 to 419. These residues corresponded to surface exposed loops. Similar dynamics of the P15-R32 loop was observed for the E266K, R267H and P300L variants, which confirmed the existence of an open state for the substrate-unbound form of hGOT1.

### Conformational dynamics of PLP

#### Hydrogen bond network and stacking interactions

**Fig 3:**
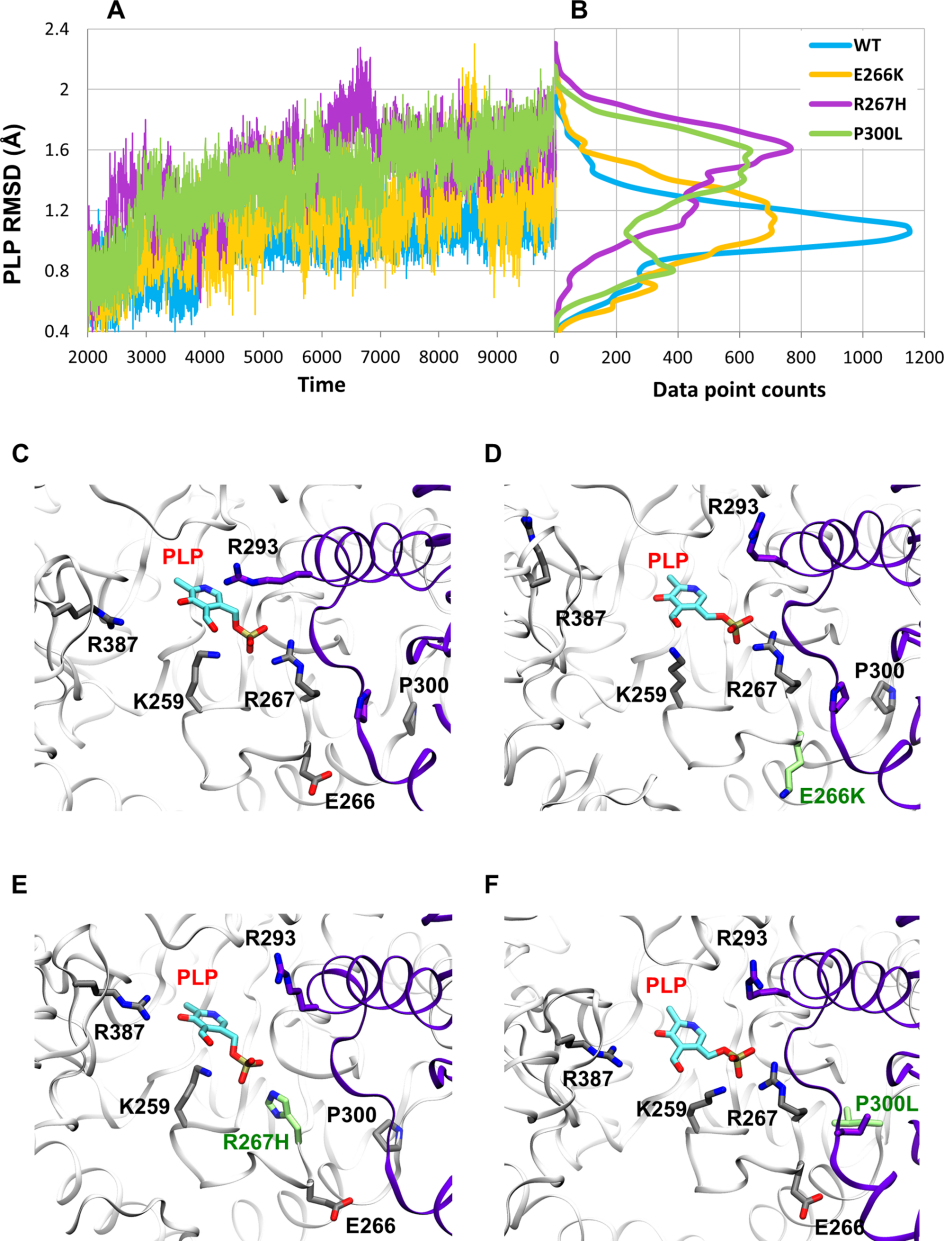
PLP dynamics in the binding pocket of hGOT1. (A) PLP RMSD values based on four 1 μs simulations of WT, E266K, R267H, and P300L. The blue plot represents WT, whereas E266K is shown in yellow, R267H is shown in purple, and P300L is shown in green. (B) RMSD distribution of PLP in each variant and WT. WT shows a much narrower distribution than the 3 variants. The color scheme is the same as in panel A. The representative PLP snapshots in the pocket are shown by (C) WT, (D) E226K, (E) R26H, and (F) P300L. The opposite monomer is colored in purple, and the amino acid substitutions are shown in green.

In order to assess PLP stability in the protein, we first compared the Root Mean Square Deviation (RMSD) values of the cofactor in the WT, E266K, R267H, and P300L systems. Fig 3A shows the PLP RMSD values as a function of simulation time and Fig 3B shows the RMSD distributions. The smallest RMSD values (0.99 Å average) and the most narrow distribution (0.19 Å) were observed for WT (Fig 3A and 3B, blue plot). Two of the variants, R267H and P300L, demonstrated significantly increased averaged RSMDs of the cofactor, 1.48 Å and 1.40 Å, respectively, as well as corresponding wider RMSD distribution of 0.27 Å and 0.29 Å.(purple and green plots in Fig 3A and 3B). The E266K variant (shown in yellow, Fig 3) also demonstrated a wide distribution of RMSD values, but slightly lower average RMSD (1.13 Å) as compared to the other two variants. These data clearly indicate that the amino acid substitutions in all three variants produced increased conformational dynamics of PLP at the binding site as compared to WT. Fig 3C, 3D and 3E show overlays of the MD snapshots of PLP at the binding site for all three variants.

**Fig 4:**
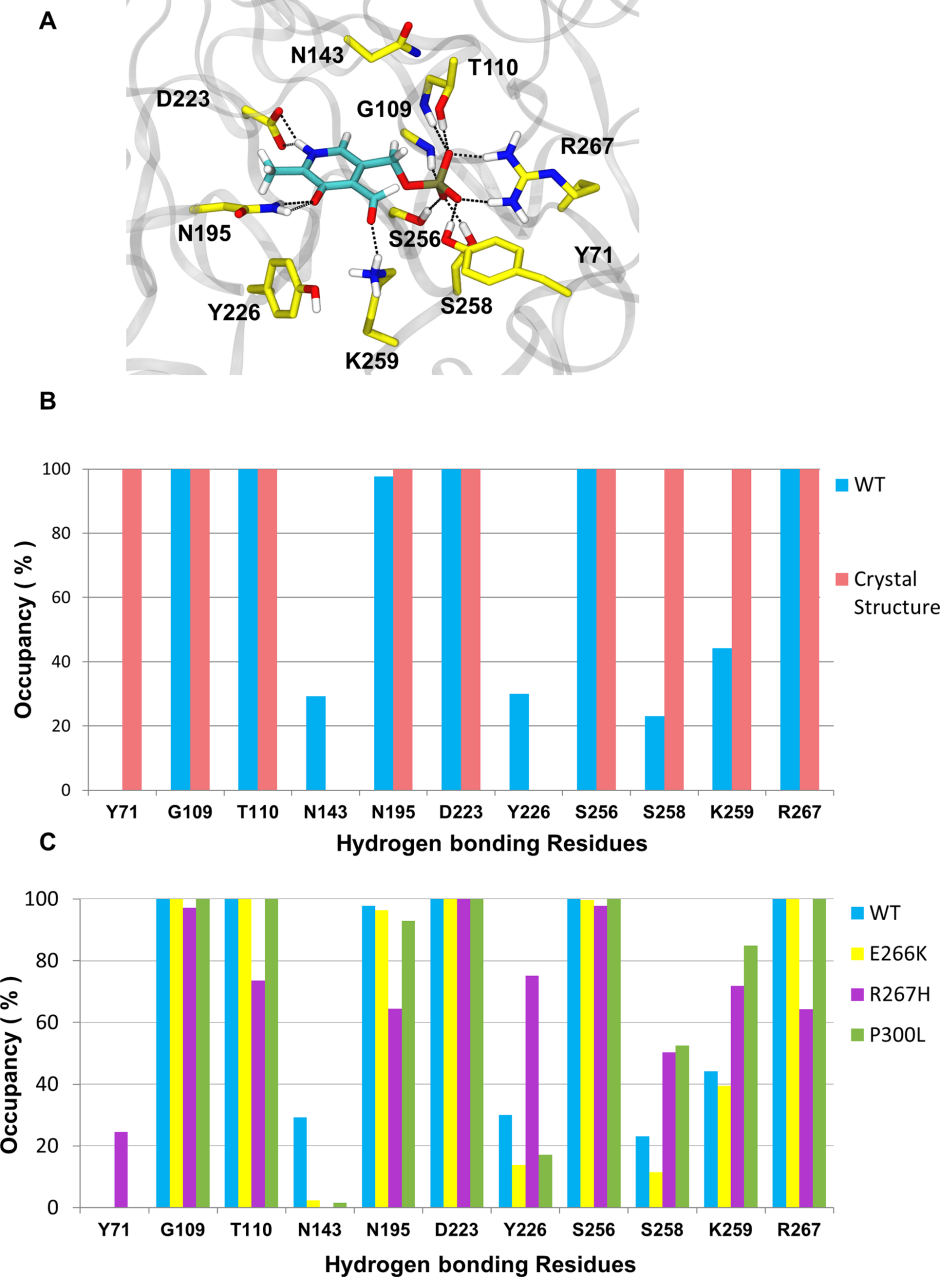
PLP coupled hydrogen bond network for WT, E266K, R267H, and P300L complexed with PLP. (A) Key residues involved in PLP stabilization in the hGOT1 binding site based on the PDB ID 3II0. (B) Refinement of the PLP hydrogen bond network in WT by MD simulation from the crystal structure. Blue bars represent the average occupancies from MD data and pink bars represent crystal data. (C) Differences in hydrogen bond occupancies between WT (blue bars), E266K (yellow bars), R267H (purple bars), and P300L (green bars) produced by MD simulation.

Other than the Schiff base, the presence and occupancy of hydrogen bonds are critical for stable non-covalent interactions between the cofactor and the enzyme. To further investigate the stability of PLP in the binding pockets of WT and variants, we analyzed the hydrogen bond network of PLP over the course of each MD simulation. Based on the WT crystal structure, the phosphate group of PLP is stabilized by the hydrogen bond network consisting of G109, T110, R267, S256, and S258 forming a polar pocket. The pyrimidine ring of PLP is anchored by hydrogen bonds between the nitrogen in the pyrimidine ring of PLP and D223, and the aldehyde of PLP and N195 and Y226 residues (Fig 4A). The WT MD simulation reproduced the occupancy of most hydrogen bonds reported in the crystal structure. Six hydrogen bonds were 100% occupied during the simulation and were identified as the key hydrogen bonds needed for stabilizing PLP binding (Fig 4B). The PLP hydrogen bonding interactions with Y71 and S258 were absent or reduced during the MD simulation indicating that these interactions could only exist in the closed conformation of the protein, and were no longer needed once the protein transitioned to its open conformation.

After identifying the essential PLP hydrogen bonding residues that contributed to stabilizing the cofactor, we compared the hydrogen bond networks between WT and the variants. In general, the hydrogen bond interactions between PLP and surrounding residues were reduced in the variants compared to WT. However, the P300L and E266K simulations revealed a similar PLP coupled hydrogen bond network observed in the WT simulation (Fig 4C). This is not surprising since this both mutations are far away from the PLP binding pocket. The mutation at the 267 position significantly altered the PLP coupled hydrogen bond network observed in the WT simulations (Fig 4C). For WT, R267 is located adjacent to the phosphate group of PLP, and makes direct contact. The replacement of R267 with the imidazole side chain of histidine reduced hydrogen bond interactions by roughly 40%. The hydrogen bond interactions between D223 and N195 with the pyrimidine ring of PLP were consistent throughout the simulation (Fig 4C). The hydrogen bonding interaction with N143 that were present in the WT simulations were not observed in all three variants.

Since most of the hydrogen bonding residues mentioned above are located around the entrance of the binding pocket, we extended our analyses of PLP stability to other important PLP stabilizing residues. Based on DFT calculations it has been shown that the PLP pyrimidine ring in the AAT pocket is stabilized by a π- π stacking interaction with W141 [6, 40]. Our WT simulation with substrate bound confirmed the near coplanar orientation between the indole ring of W144 (equivalent to W141 in AAT) and the pyrimidine ring of PLP with a distance of 3.8 Å between the planes. In the absence of substrate (open conformation of hGOT1), the average distance between the rings slightly increased to 4.4 Å. In the variants, the interactions between the PLP pyrimidine ring and W144 were reduced compared to WT. The average distances between planes increased to 5.1 Å, 6.8 Å, and 5.1 Å in E266K, R267H, and P300L simulations, respectively. In addition, the reduced π - π interactions were confirmed in all 3 variants by the loss of the coplanar orientation between the indole ring of W144 and the pyrimidine ring of PLP.

The H144, H190, and T140 residues are known to play an important role in the protonation state of the pyridine nitrogen of PLP as well as the rate of catalysis [40], yet they are not in direct contact with PLP. These residues are located on the inner wall of the PLP binding pocket within the large domain. The interactions between these residues are supported by an optimal geometry with 3Å pairwise distances between H190:T140 and T140:H144 [40]. Our WT simulation demonstrated that the distances between H190-Nε to T140-Oγ and T140-Oγ to H144-Nδ remained in the 3 to 3.3 Å range throughout the simulation. This indicated that even in the open confirmation, the binding pocket of the WT protein is structurally stable and intact. However, our variant simulations revealed significant disruption of this geometry by increasing all corresponding distances. The R267H variant displayed the largest increase in the distances between H190-Nε to T140-Oγ and T140-Oγ to H144-Nδ, reaching a maximum of 14 Å. In E266K and P300L, these distances increased to 8 Å and 10 Å, respectively. In all three variants, these displacements correlated with the loss of π- π stacking between PLP and W144. Both W144 and T140 are located on the same loop comprising part of the PLP binding pocket in the large domain. The increases of these distances indicated that conformational changes in the large domain were associated with the movement of the T140-W144 loop.

### PCA analysis and interaction energies

**Fig 5:**
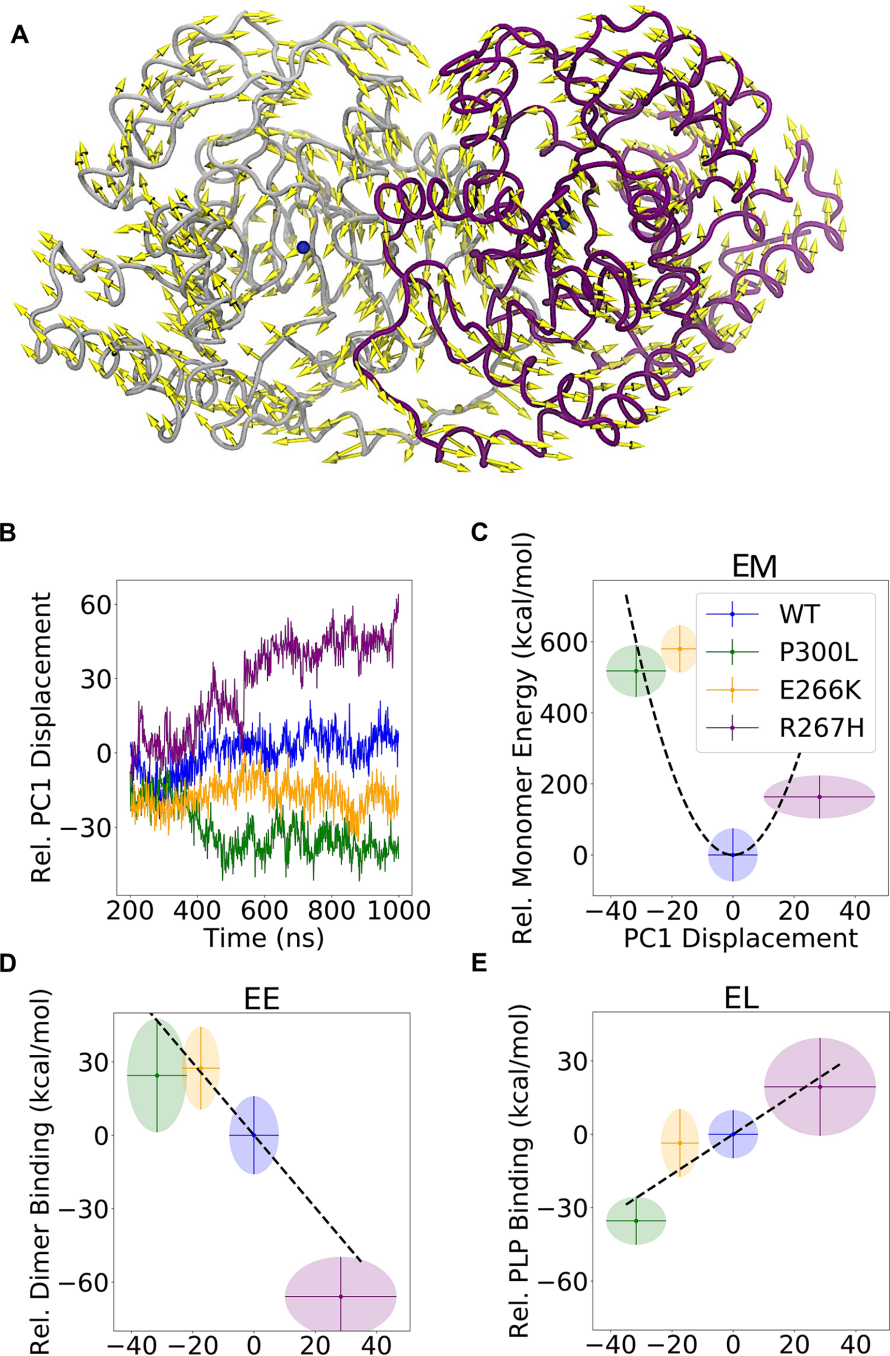
Misalignment of the hGOT1 dimer interface determined by the first principal component (PC1). A) Visualization of PC1 positive displacement (shown by yellow arrows) from PCA. Negative displacements correspond to the opposite direction of the arrows. The protein representation is the average coordinates of the inner Cα atoms (see Methods). B) The PC1 displacement of each variant as a function of simulation time. This displacement represents the magnitude of dimer interface misalignment. C) The monomer energy (EM) as a function of PC1 displacement. Ellipses show the standard deviation on both axes, and the mean values are located at the centers. D) and E) show the dimerization (EE) and PLP binding (EL) free energy versus PC1 displacement, respectively. The variants and WT follow the same color scheme as Fig 1. All values are relative to WT.

To understand the overall effect of mutations on the PLP binding and general hGOT1 stability, we performed principal component analysis (PCA) of the WT, E266K, P300L and R267H systems. PCA revealed that the largest motion described by the first principal component (PC1) resulted in misalignment of the dimer interface in all three variants compared to WT. In positive displacements, the core of the large domains (residues 80–300) rotated towards each other, while the more flexible regions (residues 40–79 and 301–315) on the opposite side of the cores rotated away from each other. The direction of the motion for positive displacement is shown as yellow arrows in Fig 5A. In negative displacements, the opposite occurred: the core of the large domains rotated outward and away from each other while the more flexible regions rotated inward. Calculating the displacements of the four simulations separately, shown in Fig 5B, revealed that WT compared to all three variants experienced no net displacement in either direction. This indicated that the dimer interface of WT was structurally intact. The R267H simulation revealed a misalignment in the dimer interface as determined by the overall large positive displacement along PC1. Misalignments in the dimer interface were also observed for the E266K and P300L variants, but were determined by an overall negative displacement along PC1. The result of this analysis suggested that the variants had a consistently misaligned dimer interface compared to WT. The largest degree of misalignment was observed for the R267H variant (Fig 5B).

Calculation of monomer energies (EM), dimerization energies (EE), and PLP binding free energies (EL) provided deeper insight into the impact of each mutation on the hGOT1 complex with PLP. Fig 5C shows that EM in all three variants was larger than WT by at least 150 kcal/mol. EE for the E266K and P300L variants was higher than WT, and followed the same pattern as EM (Fig 5D). In contrast, the dimerization energy for R267H was about 60 kcal/mol lower than WT, which suggested that misalignment of the dimer interface did not increase the dimerization energy. However, the PLP binding energy in R267H increased by about 20 kcal/mol, indicating reduced binding affinity for PLP in R267H (Fig 5E). The opposite pattern was observed in P300L, where EE increased while EL decreased relative to WT. The result of these interactions showed that for all three variants, the total energy (ET), as determined by EM, EE, and EL, was higher than WT. Table 1 shows absolute energies that were used to calculate relative values shown in Fig 5.

**Table 1:** Absolute energies calculated for WT, E226K, R267H, and P300L systems with their standard deviations. The monomer energy EM denotes the combined energy of the two monomers in the dimer, whereas EE represents the dimerization energy. PLP binding free energy is defined as EL. Both binding sites were included in the EL calculation and the sum of two are reported. The total energy (ET) in the last column is the sum of all energy components.

	EM (kcal/mol)	EE (kcal/mol)	EL (kcal/mol)	Total ET (kcal/mol)
**WT**	-42387.29 ± 106.91	-235.72 ± 25.08	-612.16 ± 32.51	-43235.17 ± 88.24
**E266K**	-41806.95 ± 95.56	-201.54 ± 17.55	-615.90 ± 35.93	-42624.39 ± 87.24
**R267H**	-42239.94 ± 75.36	-293.19 ± 20.87	-592.63 ± 54.48	-43125.76 ± 67.70
**P300L**	-41864.23 ± 108.22	-210.21 ± 27.21	-643.83 ± 16.53	-42718.27 ± 94.93

**Fig 6:**
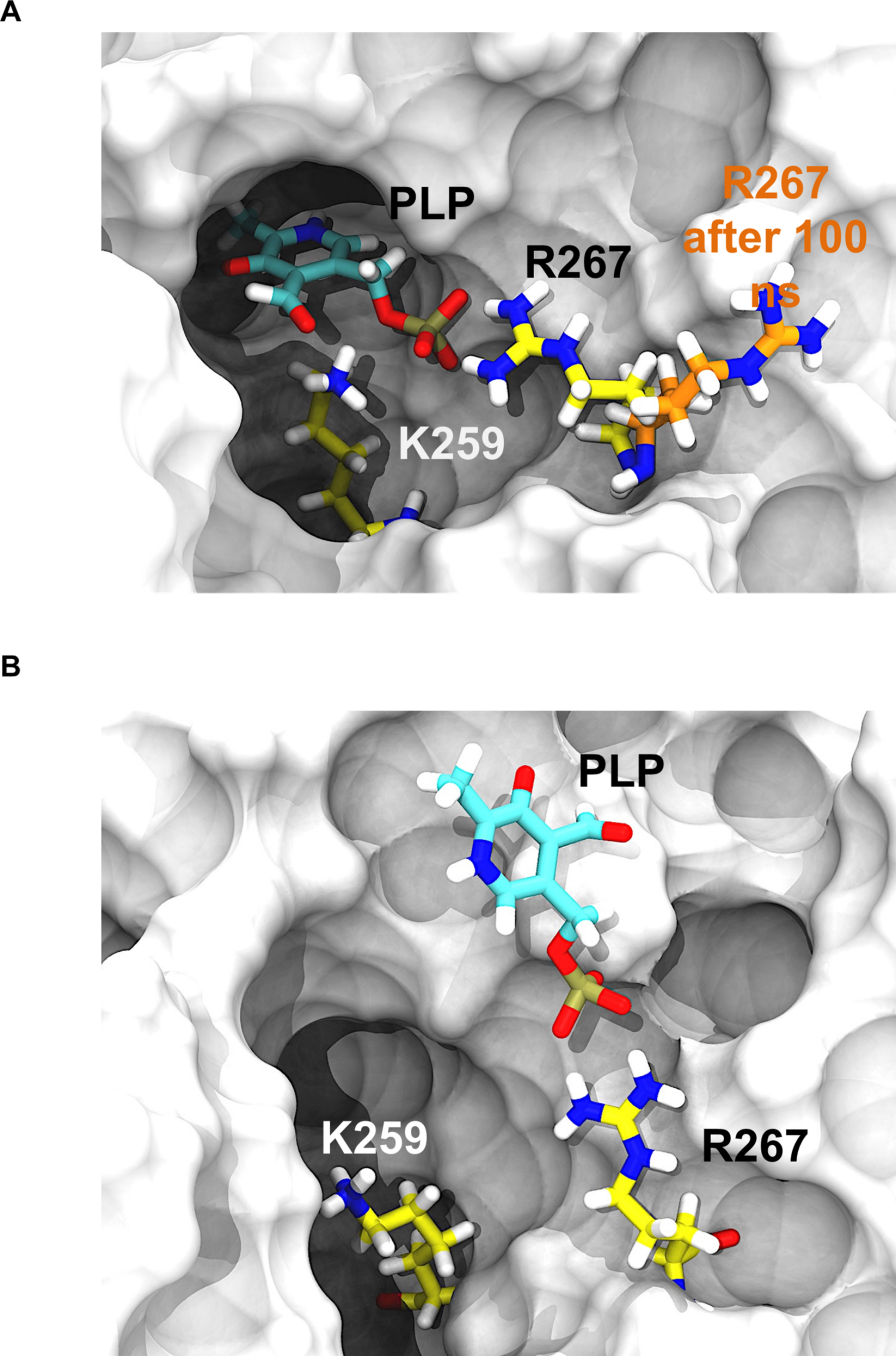
1 μs MD simulation of the hGOT1 monomer with PLP. A) Starting structure of the hGOT1 monomer showing PLP bound (colored in cyan) in the pocket (the monomer structure is based on PDB 3II0). The displacement of R267 from the PLP binding pocket observed after 100 ns of MD monomer simulation, shown as the transition from yellow to orange. B) The flipped out R267 removed essential binding interactions with PLP. The subsequent destabilization of PLP lead to its release from the binding pocket after 1 μs of MD simulation.

Modification of hGOT1 at the 266, 267 and 300 positions clearly lead to an overall unstable dimer as compared to WT. The EM, EE, and EL energy differences of the variants correlated with motions that directly affected the dimer interface and the active site. In the case of E266K and P300L, the two large domain cores separated, and made the dimer interface less stable. To compensate for EE increase, the monomers rearranged and formed more stable temporary interactions with PLP as the dimer separated (Fig 5D and 5E). However, this improved binding to PLP in P300L should be short lived since our monomer-only simulation failed to keep PLP bound in 1 μs simulation time (Fig 6). In this monomer-only simulation, the first event that lead to PLP escape was disassociation with R267. This residue was able to fluctuate and unbind PLP since the other monomer was not present to lock R267 in place. This suggests that separation of the dimer interfaces primarily responsible for PLP release in all variants.

From the dynamics observed by the R267H variant, the large domains shifted closer together, which resulted in a lower EE than WT. This was at the expense of higher EL, but this difference was largely due to the fact that the primary PLP binding residue (R267) was replaced with a residue that cannot interact as strongly. To compensate for this missing interaction, we observed that R293 of the substrate binding motif, which is present on the large domain core, moved closer to PLP and interacted with its phosphate group. This residue is primarily used for binding the substrate.

**Fig 7:**
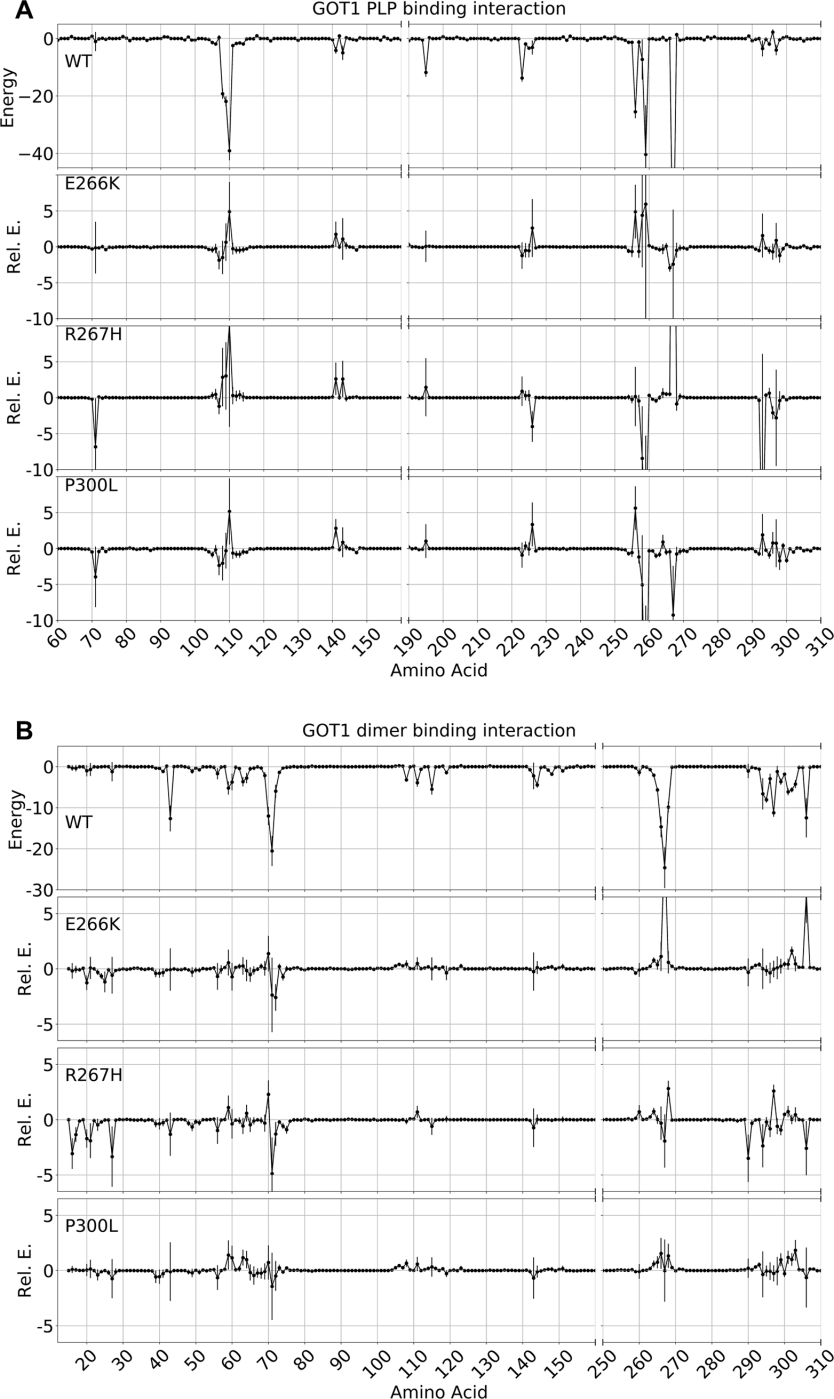
Residue pairwise decomposition of interaction energies (kcal/mol) of the dimer interface and PLP binding. To clarify the difference between WT and variants, the values for the E266K, R267H, and P300L were plotted relative to WT. Segments that have no interaction have been removed to increase the resolution of significant residues. The data represent the mean interaction energy over 800 ns, with vertical lines representing the standard deviation. A) Plots showing the individual contributions of each residue to the PLP binding energy (EL). B) Plots showing the individual contributions of each residue to the dimerization energy (EE).

A residue pairwise decomposition of energies further showed which specific residues accounted for the differences in EM, EE, and EL. While EE and EL showed clear specific differences (Fig 7), the EM increases in the variants were spread across the entire protein and no clear pattern emerged (data not shown). The residues that favorably interacted with PLP determined by this analysis also formed highly occupied hydrogen bonds with PLP, as previously described (Fig 4). Here, however, the pairwise energy analysis provided a detailed explanation of the variants. P300L formed a stronger interaction with PLP solely due to stronger K259 and R267 interactions (Fig 7A). Oppositely for R267H, the PLP binding energy for the 267 position increased by 30 kcal/mol, but R293 compensated -10 kcal, and resulted in an overall higher PLP binding energy than WT (Fig 7A). The EE energy for R267H was lower due to changes in residues similar to those observed in WT, with no new interactions formed other than R293. For E266K, the EEenergy was higher than WT solely due to R267 and I306 (Fig 7B). The I306 residue came into contact with the bulkier K266 residue, and this interaction appeared to directly increase the dimer binding energy. In addition, the energy differences for E266K were also due in part to electrostatics. Switching the negative charge of E266 to a positive charge in K266 reversed the interactions with the negatively charged PLP, adjacent positively charged R267, and positively charged K259 compared to WT. As a result, K266 interacted unfavorably with R267 and K259, which was observed in the EE and EL pairwise interaction energies (Fig 7).

## Conclusion

Here, for the first time, we report MD simulations of WT hGOT1 and three variants. We showed that the removal of the inhibitor or substrate from the crystal structure produced an open conformation of the P15-R32 loop, which reestablished access to the active site. This demonstrated, as previously reported for other homologs, that access to the active site is controlled by transitions between the closed and open conformations of this loop. We also showed from a monomer-only simulation that the formation of the dimer interface is essential for binding PLP, and we suggest that R267 is required for PLP to bind. Furthermore, based on previously identified mutations in *Arabidopsis* AAT (an hGOT1 homolog), we tested PLP stability in three key variants that were shown to release PLP. The role of the free PLP in plant homeostasis cannot be directly compared to humans. However, our work clearly demonstrated that hGOT1 could not bind PLP effectively as result of the mutations, thus displaying the same behavior as observed in the plant model. One of the substitutions was located directly in the PLP binding site (R267H), whereas the E266K and P300L mutations were located at the dimer interface. Our molecular dynamics simulations of the three variants clearly demonstrated a general structural destabilization of PLP and misalignment of the dimer interface. Analysis of the monomer, dimerization, and PLP binding energies revealed that all three variants were higher in energy and therefore less stable than WT. We suggest that dimer interface misalignment, together with the calculated overall higher energy for all three variants, could lead to dissociation of the hGOT1 dimer and ultimate release of PLP. It is likely that any mutations that compromise the dimer interface would directly affect PLP binding.
